# Post‐exercise hot or cold water immersion does not alter perception of effort or neuroendocrine responses during subsequent moderate‐intensity exercise

**DOI:** 10.1113/EP091932

**Published:** 2024-07-06

**Authors:** Campbell Menzies, Neil D. Clarke, Christopher J. A. Pugh, Charles J. Steward, C. Douglas Thake, Tom Cullen

**Affiliations:** ^1^ Centre for Physical Activity, Sport & Exercise Sciences Coventry University Coventry UK; ^2^ College of Life Sciences, Faculty of Health, Education and Life Sciences Birmingham City University Birmingham UK; ^3^ Cardiff School of Sport & Health Sciences Cardiff Metropolitan University Cardiff UK

**Keywords:** cooling, exercise, heating, recovery

## Abstract

Post‐exercise hot (HWI) and cold (CWI) water immersion are popular strategies used by athletes in a range of sporting contexts, such as enhancing recovery or adaptation. However, prolonged heating bouts increase neuroendocrine responses that are associated with perceptions of fatigue. Fourteen endurance‐trained runners performed three trials consisting of two 45‐min runs at 95% lactate threshold on a treadmill separated by 6 h of recovery. Following the first run, participants completed one of HWI (30 min, 40°C), CWI (15 min, 14°C) or control (CON, 30 min rest in ambient conditions) in a randomised order. Perceived effort and recovery were measured using ratings of perceived exertion (RPE) and the Acute Recovery and Stress Scale (ARSS), whilst physiological responses including venous concentrations of a range of neuroendocrine markers, superficial femoral blood flow, heart rate and rectal temperature were measured. Exercise increased neuroendocrine responses of interleukin‐6, adrenaline and noradrenaline (all *P <* 0.001). Additionally, perceptions of overall recovery (*P <* 0.001), mental performance capacity (*P =* 0.02), physical performance capability (*P =* 0.01) and emotional balance (*P =* 0.03) were reduced prior to the second run. However, there was no effect of condition on these variables (*P* > 0.05), nor RPE (*P =* 0.68), despite differences in rectal temperature, superficial femoral blood flow following the first run, and participants’ expected recovery prior to the intervention (all *P <* 0.001). Therefore, athletes may engage in post‐exercise hot or cold‐water immersion without negatively impacting moderate‐intensity training sessions performed later the same day.

## Abstract (highlights)

- **What is the central question of this study?**
  Does post‐exercise heating or cooling alter recovery and perception of effort during subsequent exercise?
- **What is the main finding and its importance?**
  Neither post‐exercise heating nor post‐exercise cooling altered perceived recovery or perception of effort during subsequent exercise, despite heating inducing large increases in blood flow to the lower limbs. Contrary to our hypotheses, neither heating nor cooling had any impact on inflammatory or neuroendocrine responses in plasma. Post‐exercise heating does not appear to exacerbate fatigue as previously suggested in other studies.

## 1 INTRODUCTION

Large volumes of moderate‐intensity exercise are a key training characteristic of high performing endurance athletes (Casado et al., 2021), with careful management of training stressors required to achieve the desired adaptation without excessive fatigue and maladaptation (Mujika et al., 2018). The relationship between stress, fatigue and adaptation is often manipulated using strategies aimed at either minimising fatigue or maximising adaptation. For example, interventions including cryotherapy, hydrotherapy and various nutritional strategies attempt to minimise residual fatigue and enable subsequent exercise to be performed at maintained or higher intensities and volumes (Peake, 2019). However, during typical moderate‐intensity training sessions, the magnitude of muscle damage or glycogen depletion is typically small (Gollnick et al., 1973; Peake et al., 2004), meaning the restorative capability of these strategies is not required following these types of training sessions. In contrast, interventions such as carbohydrate periodization, hypoxia, or heat stress are often used alongside training to increase the physiological stress and promote adaptation (Hawley et al., 2018). Moreover, to facilitate large training volumes, many endurance athletes also perform multiple moderate‐intensity training sessions on the same day (Casado et al., 2021). However, there are residual effects of performing multiple training sessions that may compromise training outcomes (Yeo et al., 2008), with an increase in residual fatigue a potential consequence of implementing interventions that increase physiological stress (Hawley et al., 2018), which may, in theory, exacerbate this response. Post‐exercise heating (Menzies et al., 2023) and cooling (Allan et al., 2021) are commonly used by athletes and influence both acute recovery and chronic adaptation through alterations in body temperature, blood flow and signalling pathways (Hyldahl & Peake, 2020). Therefore, research is required to investigate these strategies in the context of same day recovery following moderate‐intensity exercise.

Cold water immersion reduces peripheral blood flow (Mawhinney et al., 2013), which is suggested to enhance recovery through reducing oedema and inflammation, whilst also increasing central blood volume, which reduces heart rate and cardiovascular strain (Ihsan et al., 2016). Regardless of the physiological effects, an athlete's belief or expectation in the effectiveness of an intervention can also influence recovery (Hurst et al., 2020). Indeed, similar improvements in recovery have been observed to a repeated sprint protocol with a placebo versus cold water immersion, suggesting that in this context, many of the benefits of cold water immersion on recovery can be attributed to these expectation effects (Broatch et al., 2014). Therefore, whilst the restorative processes may not be required following moderate‐intensity exercise (below lactate threshold), this psychological effect may mean that cold water immersion still improves perceptions of recovery or fatigue.

The physiological stress imposed by the addition of heating to training may enhance both thermal and non‐thermal adaptations in endurance athletes (Hawley et al., 2018; Hyldahl & Peake, 2020; McIntyre et al., 2022). Exercising in the heat compromises training intensity (Keiser et al., 2015), making post‐exercise heating an attractive prospect for gaining these adaptive benefits without compromising training quality (e.g. Kirby et al., 2020; McIntyre et al., 2022). However, increases in the perception of effort resulting in a slower pacing strategy and impaired next‐day performance (Skorski et al., 2019) and reductions in training load (Stanley et al., 2015) have been observed following post‐exercise heating. These effects could be due to the release, at elevated core body temperatures (>0.5°C), of circulating neuroendocrine factors, such as interleukin‐6 (IL‐6), cortisol or catecholamines (Rhind et al., 2004), which are associated with the perception of effort and fatigue, during and in recovery from exercise (Cullen et al., 2020; Proschinger & Freese, 2019; Vargas & Marino, 2014). Indeed, following exercise in the heat, peak rectal temperature has previously been associated with subsequent increased perceptions of fatigue (Willmott et al., 2017). Therefore, as post‐exercise heating can result in increases in rectal temperature to above 39.0°C (McIntyre et al., 2022), these neuroendocrine mechanisms may negatively impact subsequent training session. However, this remains to be investigated.

Therefore, this study aimed to investigate the effects of same‐day recovery following moderate‐intensity exercise and subsequent hot or cold water immersion in trained runners. It was hypothesised that perceptions of fatigue and subsequently effort during a second moderate‐intensity exercise bout performed later the same day would be (i) increased by post‐exercise hot water immersion and (ii) decreased by post‐exercise cold water immersion. Additionally, it was hypothesised that the neuroendocrine responses to exercise would be increased by post‐exercise hot water immersion.

## 2 METHODS

### 2.1 Experimental design

This study comprised a randomised, repeated measures crossover design. Following the preliminary visit, participants attended the laboratory on three further occasions each separated by >6 days to complete the experimental visits. Experimental visits consisted of two fixed‐intensity 45‐min runs (Run‐1, Run‐2) on the treadmill at 95% lactate threshold (11.7 ± 1.1 kph) separated by 6 h of recovery, with post‐exercise temperature manipulation following Run‐1. One temperature manipulation was performed per visit and consisted of either (i) hot water immersion (HWI), or (ii) cold water immersion (CWI), or (iii) no immersion (CON), completed in a randomised order. Allocation of the order of test sessions was conducted using a randomization sequence in Microsoft Excel. During HWI, participants were immersed to the level of the sternum without their arms submerged in 39.9 ± 0.1°C water for 30 min. In the CWI condition participants were immersed to the level of the belly button in 13.7 ± 1.0°C water for 15 min followed by 15 min rest in ambient conditions (20.0 ± 1.2°C, 51.3 ± 12.7% relative humidity), whilst in the CON condition participants rested semi‐recumbent on a physio plinth for 30 min in ambient conditions (19.9 ± 0.8°C, 45.2 ± 10.0% relative humidity). The duration and depth of immersion for the hot and cold water conditions were chosen so as to be in line with cold water immersion guidelines (Machado et al., 2016), whilst allowing for the hot water condition to replicate protocols used within the literature to promote heat adaptation (McIntyre et al., 2022). Additionally, whilst the effects of hydrostatic pressure on recovery have previously been discussed (see Wilcock et al., 2006), the present study opted for the control condition to be completed in ambient air rather than thermoneutral water to be in keeping with the applied nature of this study. To control for effects of the menstrual cycle on thermoregulatory responses (Charkoudian et al., 2017), eumenorrhoeic female participants completed each visit 1–7 days after their self‐reported onset of menses (*n* = 1), whereas those taking oral contraceptive pills (*n* = 2) were studied during the 7‐day non‐active pill phase.

### 2.2 Participants

Based on the previously observed effects for perceived stress following post‐exercise heating (Skorski et al., 2019), the present study was powered a priori to detect an effect size of η^2^ = 0.15, with an α of 0.05 and an 80% power. Accordingly, 14 endurance runners and triathletes (3 female, 11 Male; age, 28 ± 7 years; height 1.73 ± 0.08 m; body mass, 67.6 ± 9.8 kg; maximal oxygen uptake (${\dot{V}}_{O_{2}\max}$), 3.4 ± 0.6 L/min) were briefed and provided written informed consent to participate in the present study. Participants were classified as Tier 2 (trained/developmental) according to framework set out by McKay et al. (2021) and self‐reported running 3–6 times per week, completing a mean weekly distance of 32 ± 9 miles, with 5 km seasons bests of 18:14 ± 01:02 mm:ss (men) and 23:11 ± 00:24 mm:ss (women). This study was approved by the Coventry University Ethics committee (P99264) and conformed to the *Declaration of Helsinki*, except for prior registration in a database.

### 2.3 Preliminary visit

Participants completed a submaximal and maximal running assessment on a motorised treadmill (Desmo Treadmill, Woodway, Waukesha, WI, USA). Briefly, this consisted of six to nine, 3‐min submaximal stages with running speed increasing by 1 km/h each stage until participants reported an RPE (Borg, 1982) of 17–18. Capillary blood samples were taken from the earlobe during a short (<1 min) break between stages, where participants straddled the moving treadmill belt, for the determination of blood lactate concentration (Biosen C‐Line, EKF Diagnostics, Cardiff, UK). Lactate threshold was defined as an increase in blood lactate concentration of 0.5 mM from baseline (Hughson & Green, 1982), with 95% of this calculated as the speed for the experimental visits to ensure similar metabolic responses between participants (Baldwin et al., 2000). After a short rest (∼10 min), the maximal running assessment commenced, which involved a 1% increase in gradient every minute until volitional exhaustion. Gas exchange variables were collected using a metabolic cart (Ultima Series PFX, Medical Graphics, Tewkesbury, UK) using 30 s moving averages, with the highest ${\dot{V}}_{O_{2}}$ value taken as ${\dot{V}}_{O_{2}\max}$ in accordance with the criteria of Edvardsen et al. (2014).

Upon completion of the maximal running assessment, participants were briefed on the approximate running intensity for their experimental visits and what was involved in the three experimental conditions. Participants were then asked to rate their expected perception of recovery following 6 h of recovery from 0 (not recovered at all) to 10 (maximally recovered) for each experimental condition.

### 2.4 Experimental protocol

Prior to the experimental visits, participants were instructed to arrive at the laboratory >2 h postprandial and adhere to the following pre‐trial instructions: refrain from strenuous exercise for 24 h preceding the trial, avoid caffeine on the day of testing, avoid alcohol the day before the trial, and avoid foods high in nitrite for 12 h preceding the trial. Participants were given 24 h diet and exercise diaries prior to their first visit, to enable replication of these for the subsequent visits.

**FIGURE 1:**
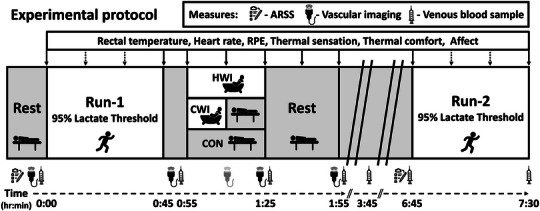
Schematic representation of the experimental protocol. Experimental conditions: HWI – 30 min, 40°C; CWI: 15 min, 14°C; CON: 30 min rest in ambient air. Tram lines (**//**) represent a break in the continuous timeline where participants were free to leave the laboratory. Light grey representation of vascular imaging denotes measure taken in CON and CWI conditions only. Dotted arrows represent time point where only heart rate and RPE were measured. ARSS, acute recovery and stress scale; CON, control; CWI, cold water immersion; HWI, hot water immersion; RPE, rating of perceived exertion.

Upon arrival at the laboratory (08.00–09.30 h), participants were given 5 mL/kg of water to standardise hydration before commencing each condition of the experimental protocol (Figure 1). Participants were able to leave the laboratory after the completion of the vascular imaging at 1:55 h:min into the experimental protocol and were asked to return for a blood sample at 3:45 h:min, and at 6:15 h:min to prepare for the Run‐2. During the period outside the laboratory, participants were instructed to replicate their diet and behavioural patterns during each experimental visit.

### 2.5 Experimental measures

#### 2.5.1 Perceptions of effort and recovery

The Acute Recovery and Stress Scale (ARSS) (Kellmann & Kölling, 2019) was completed at the start of the day and prior to commencement of Run‐2. The ARSS is split into eight subscales to assess perceptions of recovery (physical performance capability, mental performance capability, emotional balance, overall recovery) and stress (muscular stress, lack of activation, negative emotional state, overall stress) separately. Each subscale is calculated as the mean score taken from four statements about how the participant feels at the time of responding ranging from 0 – does not apply, to 6 – fully applies. Perceived exertion was measured using the 6–20 RPE scale (Borg, 1982) at 15‐min intervals and averaged (mean RPE) for each run separately.

#### 2.5.2 Thermophysiological and perceptual measures

Rectal temperature was monitored using a rectal thermometer self‐inserted 10 cm beyond the anal sphincter (Grant Instruments, Royston, UK), with simultaneous measures of heart rate (Polar FT1, Polar, Kempele, Finland). Perceptual measures of thermal sensation (+5 hot to −5 cold), thermal comfort (+5 very comfortable to −5 very uncomfortable), and basic affect (+5 very good to −5 very bad) were all recorded on modified +5 to −5 scales for ease of participant understanding (Epstein & Moran, 2006; Williams et al., 2008).

#### 2.5.3 Vascular responses

Vascular imaging of the superficial femoral artery was performed using a high‐resolution ultrasound device (T3300; Tersaon, Burlington, MA, USA). Images were obtained as soon as possible post‐exercise and immersion (∼3 min), whilst still ensuring for standardisation between participants and visits. At 1:10 h min in the experimental protocol, images were not taken in HWI condition due to the leg still being submerged. Images were recorded (∼30 s) using screen capture software (Camtasia Studio, TechSmith, Okemos, MI, UA) following optimisation of the longitudinal B‐mode image of the lumen‐arterial walls, with simultaneous Doppler velocity assessments collected using the smallest possible insonation angle (always <60°). Recordings were imported into custom‐designed edge detection and wall‐tracking software for the determination of arterial diameter and blood flow, where blood flow is calculated as lumen cross‐sectional area × Doppler velocity. This semi‐automated software is independent of investigator bias and has greater reproducibility than manual methods (Woodman et al., 2001), and in the present study had a between‐visit coefficient of variation (CV) at rest of 3.9 ± 1.9% and 27.5 ± 13.7% for diameter and blood flow, respectively. Despite this being a relatively large CV for blood flow, this was deemed acceptable given the large expected difference in blood flow between the experimental conditions.

#### 2.5.4 Blood sampling and analysis

Venous blood samples were obtained through the insertion of an indwelling cannula into an antecubital vein during the initial 2 h assessment period, with samples obtained beyond this point taken through venepuncture. Venous samples were collected into a serum vacutainer and immediately separated for the determination of blood lactate and glucose (Biosen C‐Line, EKF Diagnostics, Cardiff, United Kingdom), haematocrit (Damon/IEC Division, Needham Heights, MA, USA), and haemoglobin (Hb 201+ System, HemoCue, Ängelholm, Sweden), with the remaining blood allowed to clot before being centrifuged at 3000 *g* for 10 min. Due to sampling issues, blood samples were only collected for *n* = 12 participants. Aliquoted serum was stored at −80°C until later analysis using commercially available ELISA kits of interleukin‐6 (IL‐6) (D6050, Bio‐Techne, Minneapolis, MN, USA), the soluble IL‐6 receptor (sIL‐6r) (DY227, Bio‐Techne), adrenaline (KA3768, Abnova), noradrenaline (KA3768, Abnova, Taipei City, Taipai, Taiwan) and cortisol (KGE008B, Bio‐Techne). Samples were diluted (DY997, Bio‐Techne) in a ratio of 1:60 and 1:100 for cortisol and sIL‐6r, respectively, to ensure concentrations were within the dynamic range of each assay. To minimise variation between assays, all samples from an individual participant were analysed in the same assay. In our hands, the intra‐assay CV for these assays were as follows: IL‐6:3.6 ± 4.5%, sIL‐6R: 4.6 ± 7.6%, cortisol: 3.2 ± 2.9%, adrenaline: 3.4 ± 3.5%, and noradrenaline: 3.1 ± 2.5%. Concentrations were determined using interpolation of a four‐parameter standard curve (GraphPad Prism, GraphPad Software, Boston, MA, USA), before being adjusted for changes in plasma volume calculated according to Dill and Costill (1974).

#### 2.5.5 Statistical analysis

Data are reported as means ± standard deviation, or medians (lower quartile, upper quartile), with data on figures displaying means ± standard deviation. Statistical significance was accepted as α < 0.05. Analysis was conducted in RStudio using the functions and packages stated below. Data were examined using linear mixed effects modelling using the *lmer* function from the *lme4* package, with fixed effects including condition, time and their interaction, with random effects accounting for each individual whilst controlling for condition and time effects. The significance of fixed effects was determined using the *anova* function from the *stats* package, according to the approximations by Kenward and Roger (1997) for the denominator degrees of freedom. Data were not remodelled to other distributions, despite some violations of normality in the residuals, owing to the robustness of linear models to this violation (Schielzeth et al., 2020). *Post hoc* testing of pairwise comparisons of estimated marginal means with Bonferroni adjustment was conducted using the *emmeans* function from the *emmeans* package to evaluate differences between conditions where significant interaction effects were detected. Data from the expectancy questionnaire were analysed using Friedman's test, with *post hoc* comparisons made using pairwise Wilcoxon's test with Bonferroni adjustments using the *friedman.test* and *pairwise.wilcox.test* functions from the *stats* package.

## 3 RESULTS

### 3.1 Perceptions of effort and recovery

**FIGURE 2:**
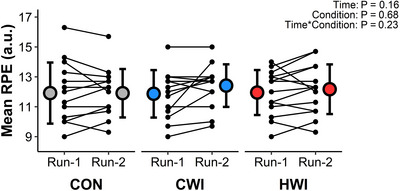
Mean RPE for Run‐1 and Run‐2 in each condition. Data were examined using linear mixed effects modelling and are presented as group means ± SD by larger coloured circles, with smaller black circles and connecting lines representing individual responses. *n* = 14.

Prior to the experimental visits, 9/14 participants rated CWI as the most effective condition for recovery (CON: 6 ± 2, CWI: 8 ± 2, HWI: 7 ± 1; *P =* 0.0002), with CWI being significantly different from CON (*P =* 0.02) but not HWI (*P =* 0.31). Despite these expected differences, mean RPE did not differ between conditions over time (Figure 2). Similarly, there was no effect of condition nor time × condition interaction for any measure on the ARSS; however, there was a time effect for all subscales of recovery but not stress with lower recovery reported prior to Run‐2 compared with Run‐1 (Table 1).

**TABLE 1:** Scores for recovery and stress from the ARSS (arbitrary units) prior to Run‐1 and Run‐2 in each condition.

	CON		CWI		HWI		
	Run‐1	Run‐2	Run‐1	Run‐2	Run‐1	Run‐2	Statistics
Recovery							
Physical performance capability	3.7 ± 1.1	3.4 ± 0.9	3.6 ± 0.6	3.3 ± 0.6	3.7 ± 0.9	3.4 ± 0.9	Time: *P =* 0.01 Condition: *P =* 0.61 Condition × Time: *P =* 0.94
Mental performance capability	3.7 ± 1.0	3.5 ± 0.7	3.7 ± 0.8	3.4 ± 0.8	3.7 ± 0.9	3.5 ± 1.0	Time: *P =* 0.02 Condition: *P =* 0.79 Condition × Time: *P =* 0.95
Emotional balance	4.2 ± 0.9	4.1 ± 0.8	4.1 ± 0.7	4.1 ± 0.7	4.3 ± 0.7	4.1 ± 0.9	Time: *P =* 0.03 Condition: *P =* 0.82 Condition × Time: *P =* 0.49
Overall recovery	4.0 ± 0.8	3.4 ± 0.7	3.7 ± 0.6	3.3 ± 0.6	3.9 ± 0.6	3.4 ± 0.6	Time: *P =* 0.0003 Condition: *P =* 0.31 Condition × Time: *P =* 0.54
Stress							
Muscular stress	2.0 ± 1.3	2.4 ± 1.0	2.4 ± 1.0	2.6 ± 1.1	2.2 ± 1.0	2.5 ± 0.9	Time: *P =* 0.07 Condition: *P =* 0.54 Condition × Time: *P =* 0.70
Lack of activation	1.7 ± 1.1	1.8 ± 1.0	1.7 ± 0.8	1.8 ± 0.8	1.6 ± 1.0	1.8 ± 1.0	Time: *P =* 0.61 Condition: *P =* 0.93 Condition × Time: *P =* 0.89
Negative emotional state	1.4 ± 1.2	1.5 ± 1.2	1.6 ± 1.2	1.6 ± 1.2	1.5 ± 1.3	1.4 ± 1.0	Time: *P =* 0.34 Condition: *P =* 0.94 Condition × Time: *P =* 0.52
Overall stress	1.4 ± 1.0	1.6 ± 1.0	1.8 ± 0.8	2.0 ± 1.0	1.6 ± 1.2	1.8 ± 1.1	Time: *P =* 0.12 Condition: *P =* 0.82 Condition × Time: *P =* 1.00

Data were examined using linear mixed effects modelling and are presented as means ± SD. *n* = 14.

### 3.2 Thermophysiological and perceptual measures

**FIGURE 3:**
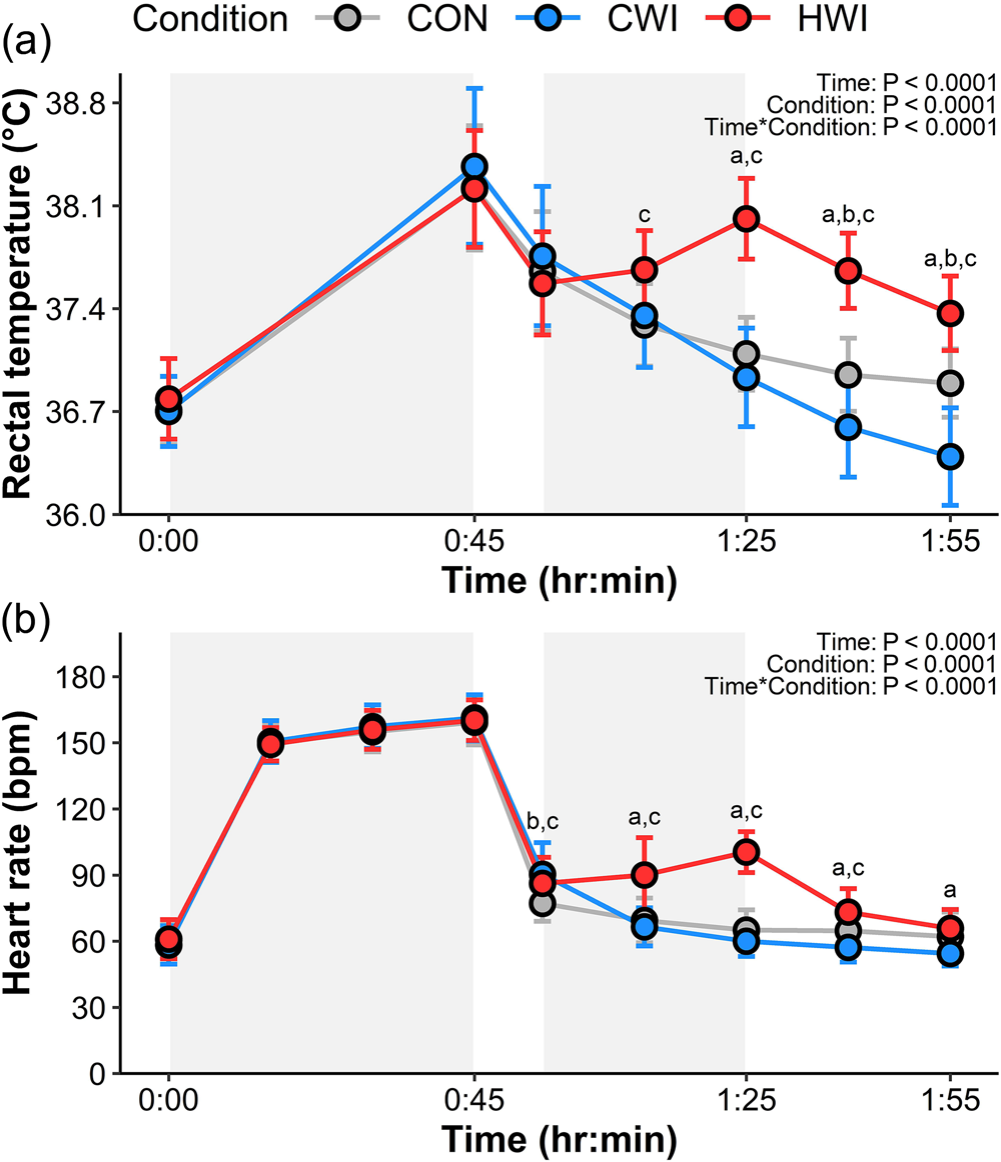
Rectal temperature (a) and heart rate (b) responses for each condition. Data were examined using linear mixed effects modelling and are presented as mean ± SD. Statistical significance is denoted as follows: ^a^HWI versus CWI, ^b^CON versus CWI, ^c^HWI versus CON. Shaded grey areas represent Run‐1 (0:00–0:45 h:min) and immersion (0:55–01:25 h:min). *n* = 14.

Rectal temperature displayed significant main effects of time, condition and their interaction (Figure 3a). During exercise, rectal temperature increased by a similar magnitude during Run‐1 and Run‐2; however, was significantly higher prior to Run‐2 (Run‐1: 36.7 ± 0.2°C, Run‐2: 37.0 ± 0.2°C; *P =* 0.04), leading to a higher peak temperature (Run‐1: 38.3 ± 0.4°C, Run‐2: 38.5 ± 0.5°C; *P =* 0.02 compared to Run‐1). Post‐exercise in the HWI condition, rectal temperature increased to 38.0 ± 0.3°C after 1:25 h:min before declining, whilst in CWI rectal temperature progressively decreased following exercise to 36.4 ± 0.3°C at 1:55 h:min. There was no difference in rectal temperature at the start of Run‐2 between HWI (37.0 ± 0.2°C) and either CWI (37.0 ± 0.3°C, *P =* 0.91) or CON (37.1 ± 0.2°C, *P =* 0.63), which were also similar (*P =* 0.71). Heart rate also showed significant main effects of time, condition, and their interaction, following a similar pattern to rectal temperature, increasing to 100 ± 11 bpm after 1:25 h:min during HWI, whilst continuing to fall post‐immersion in CWI to 54 ± 7 bpm after 1:55 h:min (Figure 3b). There was no significant difference in heart rate between Run‐1 and Run‐2 at 15 min (Run‐1: 150 ± 8 bpm, Run‐2: 156 ± 9 bpm; *P =* 0.11), 30 min (Run‐1: 156 ± 9 bpm, Run‐2: 160 ± 9 bpm; *P =* 1.00), or 45 min (Run‐1: 160 ± 9 bpm, Run‐2: 163 ± 9 bpm; *P =* 1.00).

Perceptual responses are summarised in Table 2. Differences between conditions in thermal sensation were observed only during the immersion period, with participants feeling hotter in the HWI condition and cooler in the CWI condition. During and immediately following immersion, participants reported feeling more thermally comfortable in CON compared to either CWI or HWI, however there was no difference between conditions during either Run‐1 or Run‐2. No effects of condition or time were observed for basic affect.

**TABLE 2:** Summary of thermal perception over time.

	Condition	Time (h:min)									Statistics
		0:00	0:45	0:55	1:10	1:25	1:40	1:55	6:45	7:30	
Thermal sensation (a.u)	HWI	0 (0, 0)	2.5 (1, 3)	1 (0.25, 2)	3^a^, ^c^ (3, 4)	4^a,c^ (3, 4)	0 (−0.75, 0.75)	0 (0, 0)	0 (0, 0)	2.5 (1.25, 3)	Condition: *P <* 0.0001 Time: *P <* 0.0001 Condition × Time: *P <* 0.0001
	CWI	0 (0, 0)	3 (2, 3)	1 (0, 1.75)	−2^a^, ^b^ (−2, −1)	‐0. 5^a,b^ (−1, 0)	0 (−1, 0)	−1 (−1, 0)	0 (0, 1)	2.5 (1.25, 3)	
	CON	0 (0, 0)	3 (2, 3)	1 (0.25, 2)	0^b^, ^c^ (0, 0.75)	0^b,c^ (0, 0)	0 (0, 0)	0 (0, 0)	0 (0, 0.75)	3 (2, 3)	
Thermal comfort (a.u.)	HWI	1 (0, 3)	2 (0.25, 3)	2 (0.25, 3)	2.5 (0.25, 3)	1^c^ (1, 3)	3 (0.25, 3.75)	2 (0, 3.75)	0.5 (0, 2.75)	2 (0.25, 3)	Condition: *P =* 0.02 Time: *P =* 0.49 Condition × Time: *P <* 0.0001
	CWI	2 (0, 3)	2 (0.25, 3)	1 (0, 3)	1^b^ (−0.75, 3)	1.5^b^ (0, 3)	1.5^b^ (0, 3)	1^b^ (−0.75, 3)	2.5 (0, 3)	1 (0.25, 2.75)	
	CON	1.5 (0, 3)	2 (0.25, 3)	3 (1, 3)	3 (0.5, 4)	3^b,c^ (3, 4)	3 (3, 4)	3 (3, 4)	2 (1, 3)	2 (−0.75, 3)	
Basic affect (a.u.)	HWI	3 (2, 3)	3 (1, 3)	3 (2, 3)	2.5 (1, 3)	1 (0, 3)	3 (1.5, 3.75)	3 (1.5, 3.75)	2.5 (1.25, 3.75)	2.5 (1,3)	Condition: *P =* 0.48 Time: *P =* 0.10 Condition × Time: *P =* 0.13
	CWI	3 (0.25, 3)	2 (1, 3)	2.5 (1, 3)	2.5 (1, 3)	2.5 (0, 3)	3 (0.75, 3)	3 (1.5, 3)	3 (1.5, 3)	2 (1, 3)	
	CON	3 (0.25, 3)	3 (1.25, 3)	3 (1, 3.75)	3 (1, 3.75)	3 (2.25, 3.75)	3 (2.25, 3.75)	3 (2.25, 3.75)	3 (1.25, 3)	1.5 (0,3)	

Data were examined using linear mixed effects modelling and are presented as median (lower quartile, upper quartile). Statistical significance is denoted as follows:

^a^ HWI versus CWI;

^b^ CON versus CWI;

^c^ HWI versus CON. *n* = 14.

### 3.3 Femoral blood flow and diameter

**FIGURE 4:**
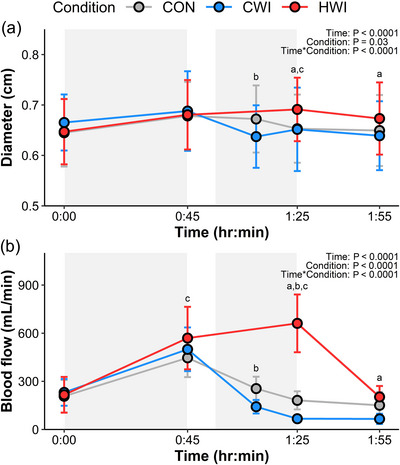
Diameter (a) and blood flow (b) responses over time for each condition in the superficial femoral artery. Data were examined using linear mixed effects modelling and are presented as means ± SD. Statistical significance is denoted as follows: ^a^HWI versus CWI, ^b^CON versus CWI, ^c^HWI versus CON. Shaded grey areas represent Run‐1 (0:00–0:45 h:min) and immersion (0:55–01:25 h:min). *n* = 14.

In the superficial femoral artery, significant main effects of time, condition, and their interaction were detected for both blood flow and diameter (Figure 4). Blood flow increased in response to Run‐1 (Δ287 ± 140 mL/min, *P <* 0.0001), with a further increase to a peak of 661 ± 180 mL/min in the HWI condition at 1:25 h:min. In both the CWI and CON conditions blood flow decreased post‐exercise to a nadir of 66 ± 32 mL/min and 151 ± 83 mL/min at 1:55 h:min, respectively. Diameter followed a similar pattern to blood flow, increasing in response to Run‐1 (Δ0.03 ± 0.04 cm, *P <* 0.0001), with a peak of 0.69 ± 0.06 cm at 1:25 h:min in the HWI condition. However, the nadir in the CWI condition of 0.64 ± 0.06 cm occurred immediately post‐immersion at 1:10 h:min before increasing to be similar to CON at 1:25 h:min (CWI: 0.65 ± 0.08 cm. CON: 0.65 ± 0.07 cm) and 1:55 h:min (0.64 ± 0.07 cm. CON: 0.65 ± 0.07 cm). In the HWI condition, both diameter (0.67 ± 0.07 cm) and blood flow (203 ± 68 mL/min) remained elevated compared to CWI min (Blood flow: *P <* 0.001. Diameter: *P =* 0.003) but not CON (blood flow: *P =* 1.0. Diameter: *P =* 0.13) after 1:55 h:min.

### 3.4 Neuroendocrine responses

**FIGURE 5:**
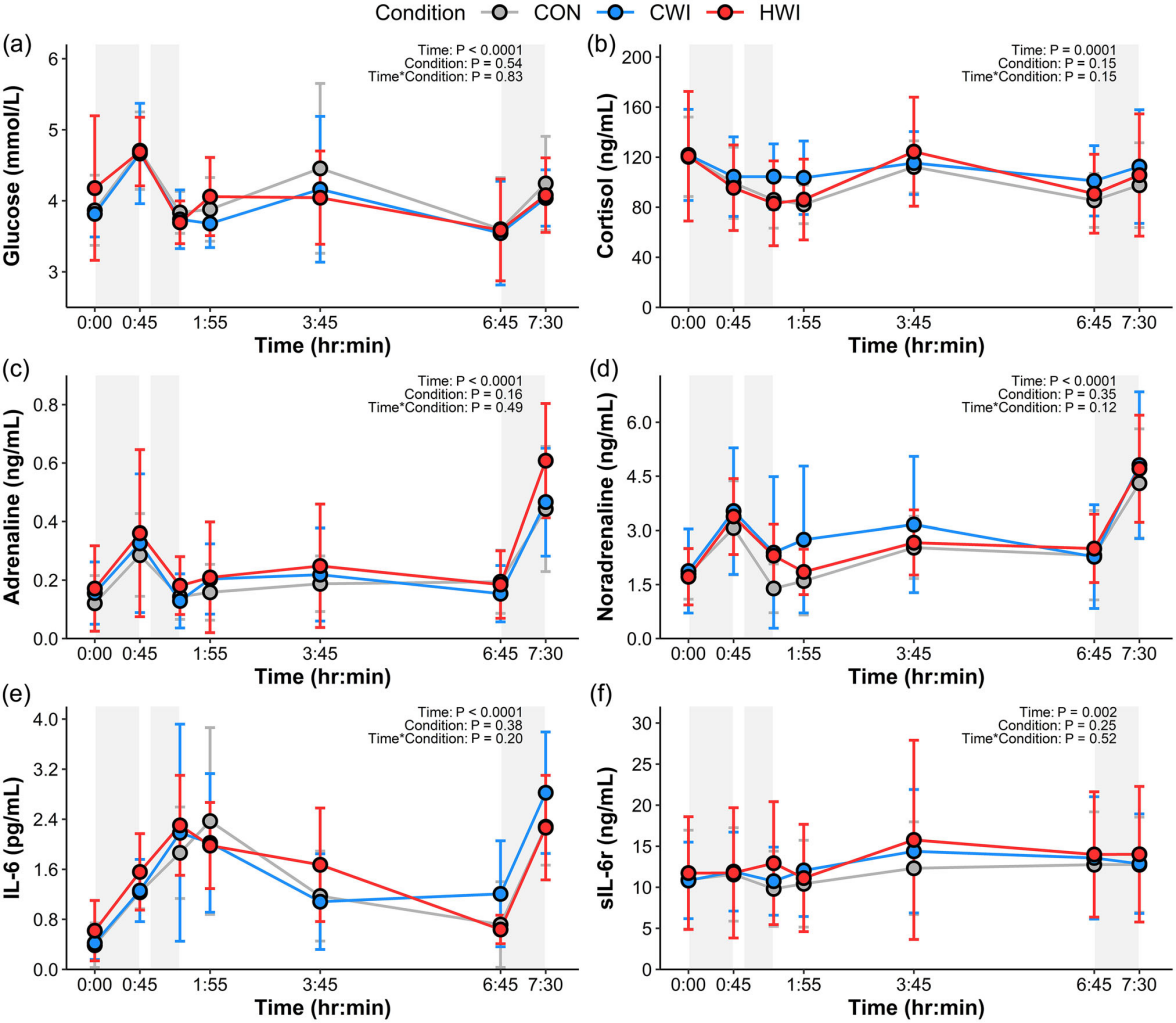
Mean ± SD responses for; glucose (a), cortisol (b), adrenaline (c), noradrenaline (d), IL‐6 (e), and sIL‐6r (f) over time for each condition. Shaded grey areas represent Run‐1 (0:00–0:45 h:min), immersion (0:55–01:25 h:min) and Run‐2 (06:45–7:30 h:min). Data were examined using linear mixed effects modelling. *n* = 12.

Blood lactate showed no significant effect of time (*P =* 0.11), or condition (*P =* 0.61) (Run‐1: Δ0.21 ± 0.41 mmol/L, Run‐2: Δ0.10 ± 0.38 mmol/L). For glucose, cortisol, IL‐6, sIL‐6r, adrenaline and noradrenaline, there was no significant effect of condition but concentrations did significantly differ over time, with no time × condition interactions (Figure 5). Prior to Run‐2, cortisol was significantly lower than before Run‐1 (Run‐1: 120.9 ± 39.8 ng/mL, Run‐2: 92.4 ± 27.4 ng/mL; *P =* 0.008), with no differences between Run‐1 and Run‐2 for concentrations of glucose (*P =* 0.57), adrenaline (*P =* 1.00), noradrenaline (*P =* 0.20), IL‐6 (*P =* 1.00) or sIL‐6r (*P =* 0.27). However post‐exercise, adrenaline (Run‐1: 0.32 ± 0.22 ng/mL, Run‐2: 0.51 ± 0.21 ng/mL; *P <* 0.0001) and noradrenaline (Run‐1: 3.3 ± 1.3 ng/mL, Run‐2: 4.6 ± 1.7 ng/mL; *P <* 0.0001) increased to a greater peak concentration following Run‐2 compared to Run‐1. Post‐exercise peak glucose concentration was greater following Run‐1 than Run‐2 (Run‐1: 4.7 ± 0.6 mmol/L ng/mL, Run‐2: 4.1 ± 0.5 mmol/L; *P =* 0.02), with no difference post‐exercise between Run‐1 and Run‐2 for concentrations of cortisol (*P =* 1.00), IL‐6 (*P =* 0.13) or sIL‐6r (*P =* 1.00). Changes in plasma volume displayed significant main effects of time (*P <* 0.0001), but not condition (*P =* 0.64), or a time × condition interaction (*P =* 0.89).

## 4 DISCUSSION

To our knowledge, this is the first study to investigate the effects of post‐exercise temperature manipulation on perceived recovery and neuroendocrine responses in the context of two moderate‐intensity training sessions performed on the same day. Exercise increased neuroendocrine responses of IL‐6, adrenaline and noradrenaline, with subsequent reductions in perceived recovery. However, contrary to our hypothesis, neither hot nor cold water immersion altered these perceptions of recovery and subsequent perception of effort during a second exercise bout performed later the same day, or the neuroendocrine responses. This was despite differences between the conditions in expected recovery, rectal temperature, heart rate and blood flow that have previously been suggested to be important in either accelerating or impairing recovery. Therefore, this study provides novel insight into the use of post‐exercise hot or cold water immersion during training and demonstrates that, in the present context, athletes engaging in post‐exercise temperature manipulation may do so without impacting subsequent fixed moderate‐intensity training on the same day. Additionally, this study showed that alterations in blood flow and increased recovery expectations are not important factors in altering subsequent perceptions of effort or recovery in this context.

Exercise reduced perceptions of recovery but not subsequent perception of effort when comparing Run‐1 to Run‐2, with neither HWI nor CWI altering this response. This may suggest that there is no acute physiological mechanism following heating that may impact subsequent training sessions. Future work may wish to expand these findings to repeated exposures to try and explain the reductions in training load previously observed during a period of post‐exercise sauna bathing (Stanley et al., 2015), which may be an artefact of the additional time requirements to complete the heating sessions rather than being physiologically driven. In support of this, McIntyre et al. (2022) showed no change in markers of over‐reaching during 12 consecutive days of post‐exercise hot water immersion. With regard to post‐exercise cooling, the present study showed increased expectations of recovery did not translate to subsequent improvements in perceived recovery or reductions in subsequent perception of effort. Indeed, perception of effort did not increase from Run‐1 to Run‐2 in the CON condition, meaning the requirements for enhanced recovery is not necessarily apparent in this context. Taken together these findings suggest that athletes seeking other benefits, such as adaptation from heating (Hawley et al., 2018; Hyldahl & Peake, 2020; McIntyre et al., 2022) or relaxation (Ahokas et al., 2019), may incorporate post‐exercise temperature manipulation within their training without positively or negatively impacting fatigue or perception of effort during subsequent training sessions on the same day.

Mechanistic underpinnings of changes to the perception of effort and recovery have previously been linked to neuroendocrine (Cullen et al., 2020; Proschinger & Freese, 2019; Vargas & Marino, 2014) and blood flow responses (Ihsan et al., 2016; Mawhinney et al., 2013). However, the present study showed no difference in RPE between Run‐1 and Run‐2 despite difference responses in adrenaline, noradrenaline and cortisol. Neuroendocrine responses are elevated following a second bout of moderate‐intensity exercise performed on the same day (Ronsen et al., 2001, 2002), with this response being linked to endogenous carbohydrate stores and circulating plasma glucose concentrations (Li & Gleeson, 2005). Therefore, as glucose concentrations were lower prior to Run‐2 than Run‐1 in the present study, this may explain the increased catecholamine response to Run‐2, with adrenaline promoting glycogenolysis to maintain the exercise intensity without impacting the perception of effort. Similarly, blood flow in the superficial femoral artery was increased by HWI and decreased by CWI, by approximately three‐fold at 1:25 h:min compared to CON (CWI: 67 ± 22 mL/min, HWI: 661 ± 180 mL/min, CON: 181 ± 56 mL/min). The mechanisms linking changes in blood flow post‐exercise to improved recovery involve increases in blood flow being linked to increases oxygen and nutrient delivery, while decreases in blood flow reduce oedema and increase central blood volume reducing cardiovascular stress (Ihsan et al., 2016). However, the present exercise protocol was designed to not induce substantial muscle damage (Peake et al., 2004) or glycogen depletion (Gollnick et al., 1973), and therefore, although not measured in this study, these restorative processes may not be required following moderate‐intensity exercise. Indeed, some research has demonstrated improved exercise performance and glycogen resynthesis after a more strenuous exercise bout (Cheng et al., 2017), and future work is required to investigate the potential positive or negative effects of post‐exercise temperature manipulations in the context of training following more strenuous exercise bouts. Nevertheless, the present study demonstrates that blood flow manipulation following moderate‐intensity exercise is not an important factor in altering responses to subsequent moderate‐intensity exercise performed later the same day, whilst changes in catecholamine responses during a second exercise bout may be linked to promoting glycogenolysis to maintain the exercise intensity without impacting the perception of effort. This finding may be particularly important given that some recovery techniques are claimed to enhance recovery by increasing muscle blood flow (Babault et al., 2011).

Despite the present study showing no negative effects of heating on subsequent perception of effort or increases in the post‐exercise neuroendocrine response, some caution is required before generalising this to other heating protocols or modalities. Indeed, higher end‐immersion rectal temperatures were reported by McIntyre et al. (2022) than the present study (∼39.2 vs 38.0°C), despite the same water temperature, a similar immersion duration, and similar increases in rectal temperature during exercise. In the present study, during the 10‐min transition from exercise to immersion, rectal temperature fell by ∼0.6°C, whilst we have previously shown non‐submersion of the arms can reduce the magnitude of increase in rectal temperature by ∼0.3°C over a 30‐min immersion (Steward et al., 2023). These differences in experimental protocol compared to arms in immersion and a ∼2 min transition time utilised by McIntyre et al. (2022) likely explain the lower rectal temperatures following immersion observed in the present study. The neuroendocrine response to heating is suggested to be exponential, with greater increases in circulating concentrations at higher core temperatures (Rhind et al., 2004). Moreover, the previously observed relationship between peak rectal temperature and the subsequent increased perception of fatigue was observed at rectal temperatures above 38.7°C (Willmott et al., 2017). Therefore, the heating stimulus in the present study may have been insufficient to impact neuroendocrine responses and may explain the lack of observed effects on subsequent perception of effort and recovery. Accordingly, whilst the present protocol was considered safe and well tolerated, the risks of thermal or orthostatic intolerance are increased by small alterations, such as immersion of the arms (Steward et al., 2023), and therefore caution is warranted in engaging in larger doses of post‐exercise heating. Furthermore, when matched for increases in core temperature, sauna‐bathing causes concomitant increases in intracranial pressure and decreases in cerebral blood flow, posing a greater challenge to the brain than hot water immersion (Gibbons et al., 2021). Accordingly, this challenge to the brain may explain some of the negative effects observed by Skorski et al. (2019) that were not seen in the present study. Finally, care was taken in the present study to ensure adequate hydration prior to commencing the experimental protocol; however, this was not measured. This may have impacted both the perception of effort (Deshayes et al., 2022) and the neuroendocrine response (Costello et al., 2018) as dehydration can increase these response, and accordingly future work should take care to control for, and measure, hydration status. Therefore, more research is required to investigate these effects of differing heating magnitudes and modalities and their impact on the neuroendocrine responses.

In summary, this study showed no effect of post‐exercise heating or cooling on same day perception of effort or recovery. This suggests athletes engaging in high volumes of moderate‐intensity training may implement these strategies seeking benefits, such as increased adaptation or relaxation, without risk of impairing recovery or increased perception of effort having a negative impact on a subsequent training session on the same day, the premise being that additional residual fatigue or increased perception of effort could lead to an athlete reducing the intensity or duration of subsequent training sessions. Moreover, in the context of moderate‐intensity training that characterises large volumes of training by endurance athletes, this study showed that alterations in blood flow and increased recovery expectations are not important factors in altering subsequent perceptions of effort or recovery in this context. Finally, caution is warranted when in engaging in larger doses of heating than used in the present study, and future work should seek to investigate how responses differ from other protocols with different combinations of exercise and heating or cooling.

## AUTHOR CONTRIBUTIONS

Campbell Menzies, Tom Cullen, Neil Clarke, Christopher Pugh and Doug Thake were responsible for the conception and design of the study. Campbell Menzies, Charles Steward and Tom Cullen were responsible for data acquisition, while all authors assisted indd interpretation of the data. All authors contributed to drafting or revision of the written work, approved the final version and agree to be accountable for all aspects of the work in ensuring that questions related to the accuracy or integrity of any part of the work are appropriately investigated and resolved. All persons designated as authors qualify for authorship, and all those who qualify for authorship are listed.

## CONFLICT OF INTEREST

None declared.

## FUNDING INFORMATION

None.

## Supporting information

Raw data

## Data Availability

Data available in the Supporting information.
